# Quantification and Metabolite Identification of Sulfasalazine in Mouse Brain and Plasma Using Quadrupole-Time-of-Flight Mass Spectrometry

**DOI:** 10.3390/molecules26041179

**Published:** 2021-02-22

**Authors:** Jangmi Choi, Min-Ho Park, Seok-Ho Shin, Jin-Ju Byeon, Byeong ill Lee, Yuri Park, Young G. Shin

**Affiliations:** Institute of Drug Research and Development, College of Pharmacy, Chungnam National University, Daejeon 34134, Korea; jangmi.choi.cnu@gmail.com (J.C.); minho.park.cnu@gmail.com (M.-H.P.); seokho.shin.cnu@gmail.com (S.-H.S.); jinju.byeon.cnu@gmail.com (J.-J.B.); byungill.lee.cnu@gmail.com (B.i.L.); yuri.park.cnu@gmail.com (Y.P.)

**Keywords:** CNS, sulfasalazine, brain to plasma ratio, LC-ESI-TOF-MS

## Abstract

Sulfasalazine (SAS), an anti-inflammatory drug with potent cysteine/glutamate antiporter system xc-(SXC) inhibition has recently shown beneficial effects in brain-related diseases. Despite many reports related to central nervous system (CNS) effect of SAS, pharmacokinetics (PK) and metabolite identification studies in the brain for SAS were quite limited. The aim of this study was to investigate the pharmacokinetics and metabolite identification of SAS and their distributions in mouse brain. Using in vivo brain exposure studies (neuro PK), the PK parameters of SAS was calculated for plasma as well as brain following intravenous and oral administration at 10 mg/kg and 50 mg/kg in mouse, respectively. In addition, in vivo metabolite identification (MetID) studies of SAS in plasma and brain were also conducted. The concentration of SAS in brain was much lower than that in plasma and only 1.26% of SAS was detected in mouse brain when compared to the SAS concentration in plasma (brain to plasma ratio (%): 1.26). In the MetID study, sulfapyridine (SP), hydroxy-sulfapyridine (SP-OH), and N-acetyl sulfapyridine (Ac-SP) were identified in plasma, whereas only SP and Ac-SP were identified as significant metabolites in brain. As a conclusion, our results suggest that the metabolites of SAS such as SP and Ac-SP might be responsible for the pharmacological effect in brain, not the SAS itself.

## 1. Introduction

Drug development for central nervous system (CNS) disorders has many hurdles in terms of brain penetrability due to the inherent physicochemical properties of drugs and the complex environment such as brain-blood barrier (BBB) [1,2]. Despite the challenges of discovery for new targets and mechanisms of CNS diseases, the current treatment strategies for CNS diseases are mostly limited to the modulation of disease-derived symptoms. One of the recent approaches was that inhibition of cysteine/glutamate antiporter system xc-(SXC) could be a therapeutic potential for CNS disorder in respect of regulating glutamate concentration in glial cells [3,4,5].

**Figure 1:**
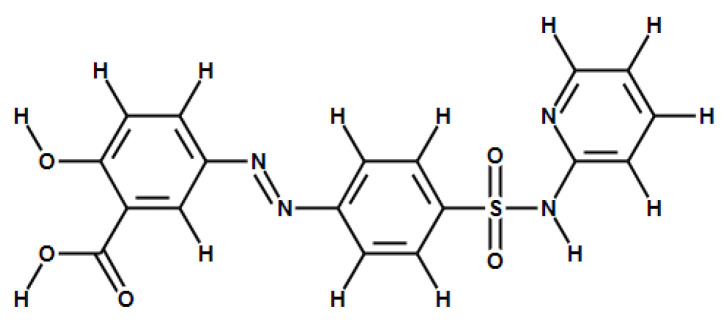
Chemical structure of sulfasalazine.

Sulfasalazine (SAS, Figure 1) is broadly used to treat chronic inflammation in the gut, joints, and retina [6]. The mechanisms that explain anti-inflammatory activity may involve the inhibition of NF-κB signaling pathway [7]. In addition, SAS has also shown clinical potential as an effective inhibitor of xc-transporter, which has a potential as a new therapeutic approach for CNS diseases [8,9,10]. Recently, some reports suggested that the effectiveness of SAS in vitro and in vivo model of brain tumor was likely due to the inhibition of the NF-κB signaling pathway by SAS [7]. Other studies have shown that SAS may contribute to the antidepressant-like effect because of its SXC inhibitory effects [10]. Another recent study explored that SAS was able to decrease the duration of epileptiform events associated with the modulation of glutamate system, which decrease the release of excitatory glutamate in the brain [11]. Therefore, due to its wide range of effects, SAS has been investigated in a number of preclinical and clinical studies [9,12,13,14,15,16,17]. Unfortunately, despite the fact that SAS has been studied recently in the areas of CNS-related research field, no/little information regarding drug metabolism and pharmacokinetics (DMPK) of SAS itself nor its metabolites in the brain have been reported.

In this study, the quantification and the metabolite identification for SAS in mouse brain and plasma have been simultaneously explored to link the effects of SAS in the CNS-related disease models. Generally, in vivo brain exposure studies (a.k.a.; neuroPK) are involved with assessing the extent (Kp_brain_) of partitioning into the brain from the blood [2]. Therefore, Kp_brain_ was calculated to evaluate the permeability of SAS passing through the BBB from its CNS effect perspectives [18,19,20,21,22]. In addition, in vivo metabolite identification (MetID) was conducted to investigate SAS and its metabolites in mouse plasma as well as in mouse brain. To our best knowledge, this is the first report regarding SAS exposure and metabolite identification in mouse brain. Hydrogen/deuterium exchange (H/D exchange) for the product ion spectra by the electrospray ionization (ESI) was also performed to confirm the fragment pattern of SAS [23,24].

## 2. Results

### 2.1. Method Development and Qualification

The liquid chromatography-electrospray-time-of-flight mass spectrometry (LC-ESI-TOF-MS) method was newly developed for SAS in mouse plasma over concentration ranges of 9.15~6670 ng/mL, as shown in Figure 1. Successive linear calibration curves were obtained over these concentration ranges with average correlation coefficients >0.99. The lower limit of quantification (LLOQ) was 9.15 ng/mL using the simple protein precipitation method.

Accuracy was evaluated at three different levels of quality control (QC) samples over three run times. There were three replicates of 15.0, 165, and 1820 ng/mL evaluated for the accuracy calculations. The inter-run accuracy ranged from 94.2% to 114% and the intra-run accuracy ranged from 98.7% to 109%, respectively. These results fall within the acceptance criteria internally made for this type of the fit-for-purpose research (±25% of the nominal value for the accuracy and precision). Precision was also evaluated at three different levels of QC samples over three run times. The intra-run precision ranged from 1.10% to 10.0% and the inter-run precision ranged from 4.09% to 8.71%, respectively. The precision data for both intra and inter-run did not exceed 10.01% from the nominal values and were less than the accepted limit of 25% CV. The repeated injection of LLOQ was also performed to assess the assay performance.

All QC samples met the acceptance criteria for this fit-for-purpose research study within ± 25% of the nominal value and the results are summarized in Table 1 and Table 2. The accuracy and precision of LLOQ were also 111% and 13.0%, respectively, as shown in Table 3. In addition, Table 4 shows that the accuracy and precision of dilution QC were also 88.4% and 13.9%, respectively.

### 2.2. Pharmacokinetic Study–Kp_brain_

**Figure 2:**
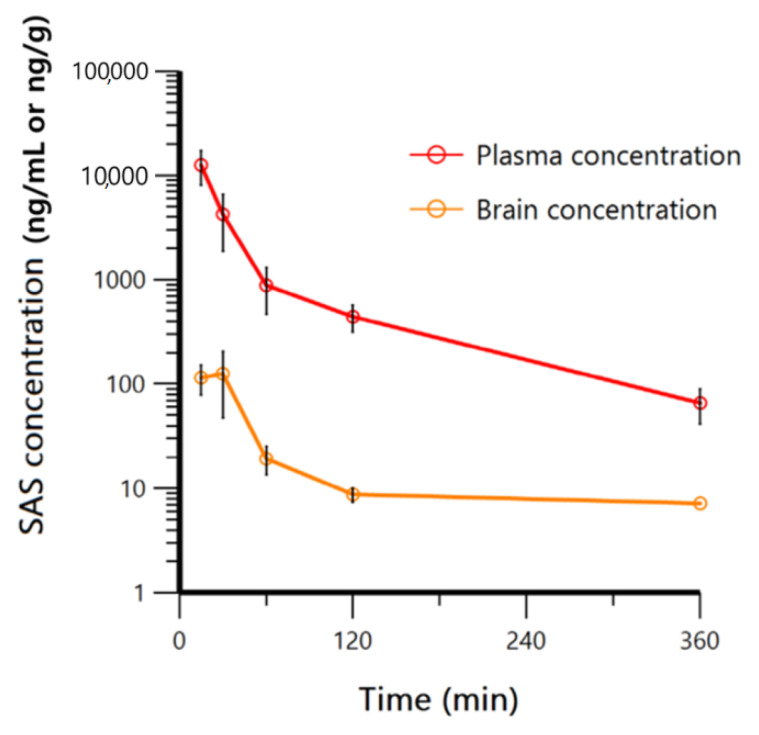
Time-concentration profiles of sulfasalazine for pharmacokinetics (PK) study (IV: 10 mg/kg) sample (plasma (red line) and brain (yellow line)).

The developed LC-ESI-TOF-MS method was successfully applied to determine the PK parameters after intravenous (IV) administration at singly dose of 10 mg/kg for SAS in mice (Figure 2). Both plasma and brain homogenates were used to determine brain to plasma ratio (%) of SAS in assessing the kinetics of drug penetration across the BBB. While most of the plasma samples were within the range of the qualified calibration curve (range: 9.15–6670 ng/mL), some samples were diluted with blank mouse plasma for covering the higher concentration over the upper limit of quantification (ULOQ), particularly for the early time point samples. Final plasma concentrations were calculated by considering the dilution factors for 5 × and 10 × of the measured concentrations for those samples above the ULOQ.

For the brain homogenate sample analysis, the evaporation and reconstitution process was used to increase the sensitivity due to relatively poor SAS exposure in brain. Furthermore, the surrogate-matrix mixing with blank mouse plasma to the brain homogenates was used to reduce the unknown non-specific binding, as well as matrix effect during electrospray ionization for LC-ESI-TOF-MS analysis [25]. Although the intra/inter run assays for SAS in brain homogenates have not been fully performed, the calibration curve samples and QC samples for the brain homogenates were prepared freshly to minimize any stability issues during the brain homogenate sample analysis. The regression of brain homogenate standard curve was also acceptable with the regression coefficient (0.998) in the range of 3.05–2200 ng/mL and the accuracies of brain homogenate QC samples were 92.2~106%. It would be ideal to evaluate several stability tests and matrix effects in each matrix to demonstrate the robustness of this assay but the stability issue was not significant due to the fresh sample preparation in this study for the brain homogenate sample analysis. Furthermore, no significant internal standard peak response variation was observed during the entire sample analysis, which would be able to explain to some extent that the matrix effect was probably not significant. In addition, the same matrix used for each calibration curve (e.g., plasma calibration curve for the plasma samples and the brain homogenate calibration curve for the brain homogenate samples) would be also able to help to normalize the matrix specific peak response, if any. Final brain concentrations were calculated by considering the dilution factors 4 x of the measured concentrations. Upon obtaining concentration-time profile data of plasma (ng/mL) and brain (ng/g), the non-compartmental analysis (NCA) using WinNonlin (version 8.0.0) was performed to estimate the PK parameters for SAS, as presented in Table 5.

PK results have demonstrated the volume of distribution (Vd) and clearance (CL) calculated from plasma concentration of 1740 mL/kg and 14.8 mL/min/kg, respectively, showing that SAS has a low clearance in mice. The area under the curve up to last time point (AUC_last_) for plasma and brain were 674,000 and 8460 min × ng/mL, respectively. The brain to plasma AUC ratios (Kp_brain_) were then calculated to evaluate the efficiency of SAS passing through the brain. Kp_brain_ of SAS was calculated to be 1.26%. These results suggested that SAS, as an intact form, would hardly penetrate the BBB, which is challenging in correlating the role of SAS for the CNS-related disease studies. Instead, this result indicated that the SAS efficacy against CNS-related studies in vivo might be likely due to its metabolites in brain or other indirect modes of actions in vivo. To understand its clinical efficacy of SAS, a metabolite identification (MetID) study was designed and conducted in mice to investigate the metabolism of SAS in mouse brain.

### 2.3. In Vivo Metabolite Identification

#### 2.3.1. Collision-Induced Dissociation (CID) Analysis

The collision-induced dissociation (CID) patterns of SAS were accomplished by LC-ESI-TOF-MS analysis. High resolution-electrospray ionization (ESI) mass spectra were obtained to determine exact masses of ions and to identify the mass fragment patterns of the compounds for the MetID study. H_2_O and D_2_O solvents were also used for the H/D exchange study to better understand unknown fragments. The fragmentation patterns of SAS obtained with both H_2_O and D_2_O solvent infusion methods were similar, however, some differences after the H/D exchange were also observed. Particularly the fragmentation using D_2_O solvent showed some different fragment ions, which seem to be related to the neutral losses of SAS [23,24]. SAS has a molecular ion at *m*/*z* 399.0772 and 403.0993 with H_2_O and D_2_O solvent, respectively, and this result matches well with the number of exchangeable protons (*n* = 3) in SAS. The product ion scan of *m*/*z* 399.0772 with H_2_O leads to the formation of fragment ions at *m*/*z* 94.0533, 119.0132, 147.0193, 165.0298, 223.0511, 315.0889, 317.1047, 333.0996, and 381.0663. However, the product ion scan of *m*/*z* 403.0993 with D_2_O leads to the formation of fragment ions at *m*/*z* 119.0138, 167.0435, 315.0901, 319.1169, 335.1117, and 383.0789. The presence of common fragment ions at *m*/*z* 119.0132 (119.0138 in D_2_O) and 315.0889 (315.0901 in D_2_O) suggests that these fragments were not subjected to H/D exchange. For fragment ions of *m*/*z* 165.0298, 317.1047, 333.0996, and 381.0663 from the product of ions of 399.0772, two amu-increased fragment ions were observed at *m*/*z* 167.0435, 319.1169, 335.1117, and 383.0789 from the product of ions of 403.0993. Especially, the observation of a fragment ion at *m*/*z* 333.0996 from the product ion of *m*/*z* 399.0772 was quite interesting because it matched with the direct loss of sulfoxylic acid (H_2_SO_2_) from SAS based on the calculation of its neutral loss by high resolution mass. This was also confirmed by monitoring a unique fragment ion at *m*/*z* 335.1117 from the product ion spectrum of SAS with D2O, which indicates the loss of equivalent neutral ion with deuterium (D_2_SO_2_).

#### 2.3.2. Brain Distribution

**Figure 3:**
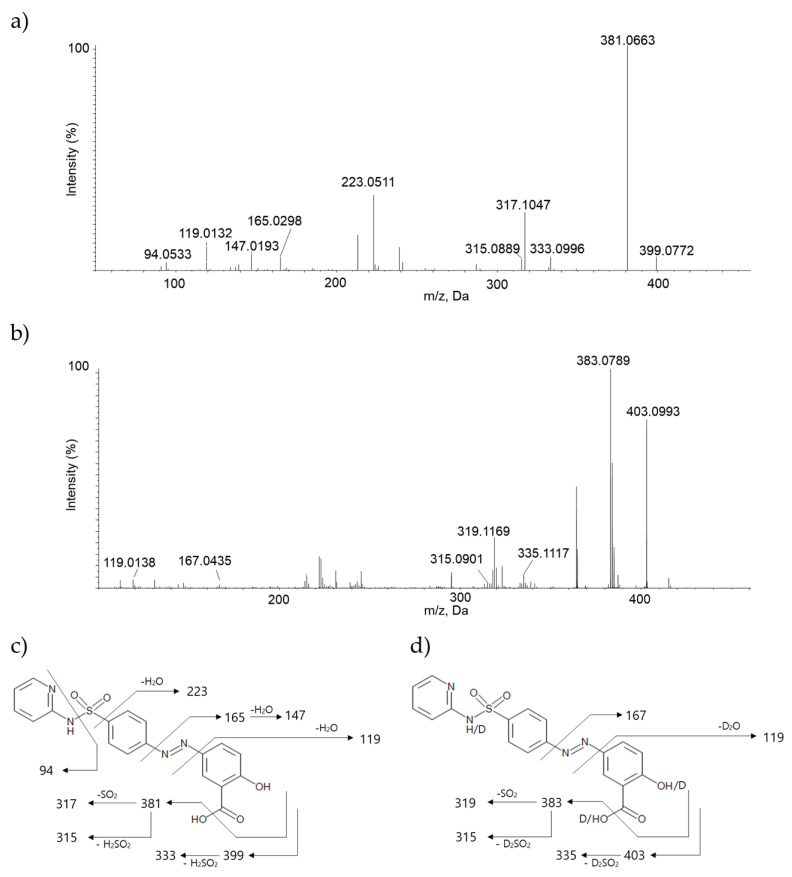
Comparison of the product ion spectra of sulfasalazine (SAS) infused in (**a**) H_2_O solvent, (**b**) D_2_O solvent, fragment pattern of SAS in (**c**) H_2_O solvent and (**d**) D_2_O solvent (representative *m*/*z* of each fragment was rounded off to zero decimal place).

**Figure 4:**
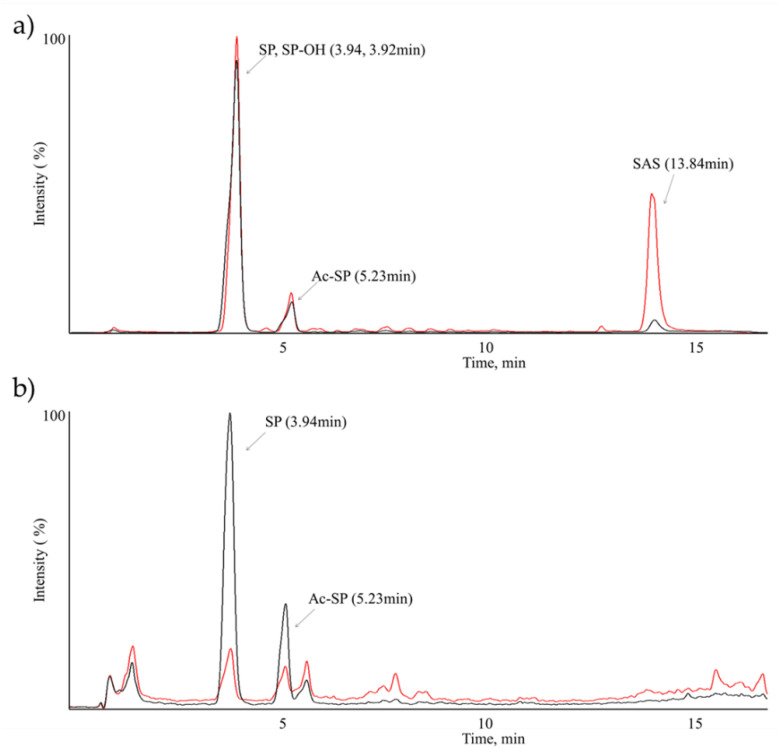
Extracted ion chromatograms (XIC) of SAS MetID samples in mouse (**a**) plasma and (**b**) brain (IV 10 mg/kg (Red line) and PO 50 mg/kg (Black line)).

**Figure 5:**
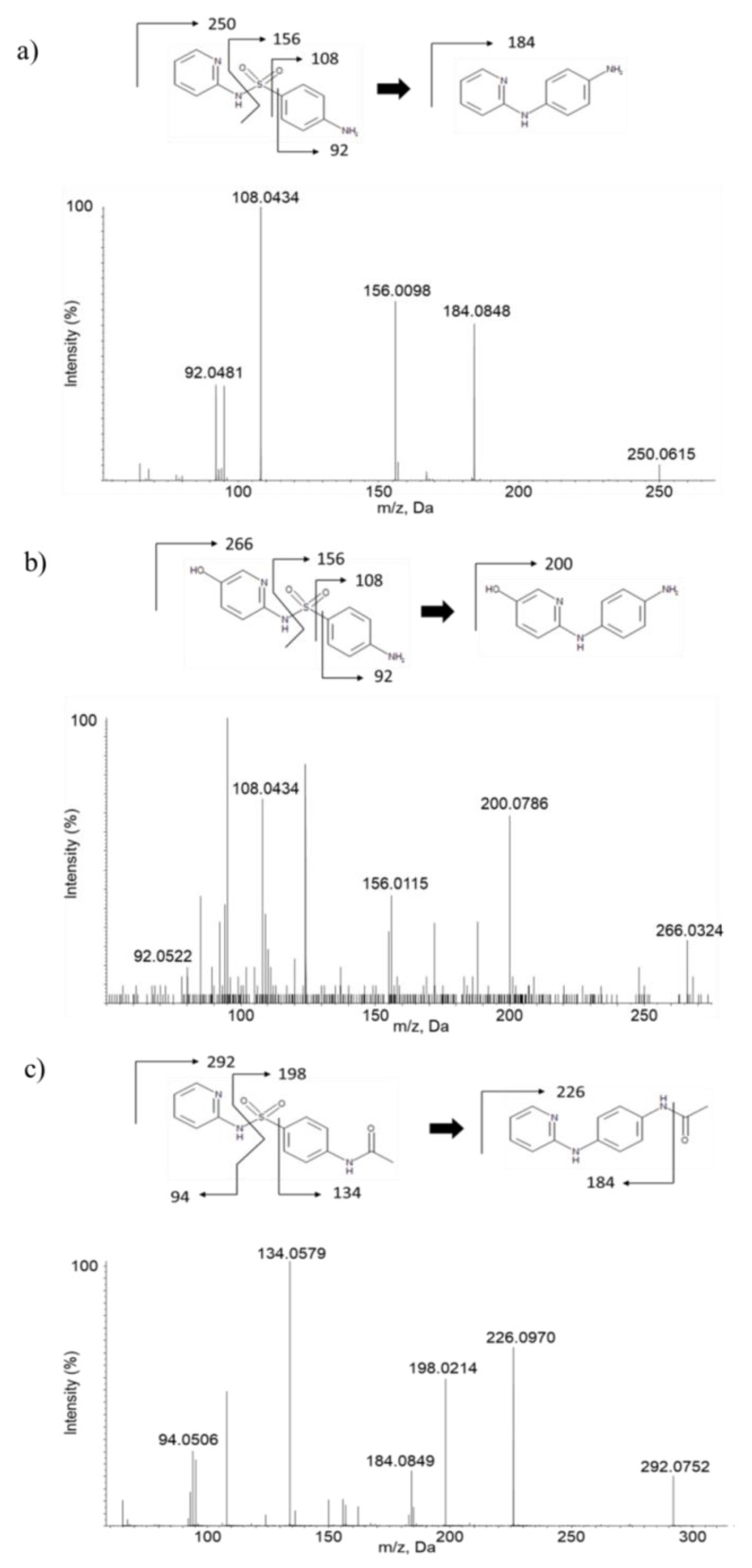
Product ion spectra of in vivo metabolites of SAS; (**a**) Sulfapyridine (SP), (**b**) Hydroxy-sulfapyridine (SP-OH), and (**c**) N-acetyl sulfapyridine (Ac-SP) in mouse plasma and brain (representative *m*/*z* of each fragment was rounded off to zero decimal place).

Representative extracted ion chromatogram (XIC) and the product ion spectra of metabolites from SAS from mouse plasma and brain are shown in Figure 3, Figure 4 and Figure 5. Under our experimental conditions, at least three significant metabolites (SP, SP-OH and Ac-SP) were detected and characterized in vivo from mouse plasma and two significant metabolites SP and Ac-SP were detected and characterized in vivo from mouse brain after 10 mg/kg IV and 50 mg/kg PO administration. Additionally, as shown in Figure 4, the metabolite peak intensity from SAS following the IV and PO administration confirmed that no/little SAS as intact was present in the brain, whereas another two metabolites were detected to significant levels based on their intensities. There seemed no significant difference in terms of metabolite profiles between IV and PO samples as well as plasma and brain samples, except the significantly low level of SAS in brain regardless of administration. Sulfasalazine is normally administered orally, therefore the CNS-related efficacy of SAS in preclinical or clinical studies might be likely due to its metabolites, not the intact SAS based on this experiment.

#### 2.3.3. In Vivo Metabolites Identification

For LC-ESI-TOF-MS analysis of SP, the precursor ion of *m*/*z* 250.1 leads to the formation of fragment ions at *m*/*z* 92.0481, 108.0434, 156.0098, and 184.0848. For LC-ESI-TOF-MS analysis of SP-OH, the precursor ion of *m*/*z* 266.1 leads to the formation of fragment ions at *m*/*z* 92.0522, 108.0434, 156.0115, and 200.0786. The unchanged fragment ion *m*/*z* 156.0115 suggests that metabolism has occurred in the pyridine ring moiety of SAS. The product ion at *m*/*z* 200.0786, which is 16 amu higher than SP, suggests the metabolism of hydroxylation to SP has occurred. For LC-ESI-TOF-MS analysis of Ac-SP, precursor ion of *m*/*z* 292.1 leads to the formation of fragment ions at *m*/*z* 94.0506, 134.0579, 184.0849, 198.0214, and 226.0970. The unchanged fragment ion *m*/*z* 184.0849 suggests that metabolism has occurred in the amide group in phenyl ring moiety of SAS. The product ions at *m*/*z* 134.0579, 198.0214, and 226.0970 were 42 amu higher than the SAS fragments at *m*/*z* 92, 156, and 184. These results explained that Ac-SP is a N-acetylation metabolite of SP. All metabolites were also compared and confirmed with the authentic standards after the LC-ESI-TOF-MS analysis (Table 6).

## 3. Discussion and Conclusions

SAS is an anti-inflammatory and immune-modulating drug that has been used for rheumatology and inflammatory bowel disease. In addition to the commonly known inflammation related efficacy, research on the CNS-related effect of SAS has recently been carried out [9,10,18,21,22]. Previous studies revealed that orally administered SAS exerted the antidepressant-like effects that were at least as effective as fluoxetine [10]. In addition, concomitant of its major metabolites, 5-ASA and SP also showed a similar tendency [10]. Nevertheless, whether SAS and its metabolites can penetrate brain or not has not been studied so far. Thus, in this paper, in vivo brain exposure studies and brain MetID study were conducted to investigate the pharmacokinetics and brain distribution of SAS in a mouse model.

As a result, the finding of poor brain-penetration of SAS as 1.26% suggests that in vivo CNS activity of SAS is most likely not directly correlated with SAS itself but with other opportunities of metabolites activity or other unknown indirect mode of actions. According to previous studies of SAS metabolism and distribution, SP is relatively well absorbed from the intestine and mainly acetylated and conjugated as a glucuronide in the liver before excretion in the urine, whereas another metabolite 5-ASA is minimally absorbed and passes out in the feces [26]. Furthermore, highly bound to albumin (>99%) of SAS is reported whereas SP is only 70% bound to albumin. In conclusion, these results suggested that the probability of SAS in alleviating CNS symptoms, depressant, and seizures is possibly due to its presence of metabolites, not SAS itself in the brain.

Although the metabolism of SAS has been studied for a long time, the BBB penetration of SAS as well as the metabolism of SAS in brain has not been reported so far. In this MetID study, SP, SP-OH and Ac-SP were identified in plasma, and SP and Ac-SP were identified in brain, regardless of drug administration route. After PO administration, SAS is metabolized by gut bacterial azo-reduction in the colon, while liver-azoreductases serve cleavage of SAS after IV administration. The metabolites of SP, SP-OH and Ac-SP following IV administration of SAS are evidence that human azoreductases, which play a crucial role in the metabolism of SAS [27,28]. In addition, SP and its secondary metabolites, Ac-SP, were only observed in brain among the significant metabolites generated by azo-reduction. These results imply that there might be some differences in terms of gastrointestinal absorption of metabolites after azo-reduction in the gut.

SAS is still being studied in the CNS-related diseases by SXC inhibitory effects. Despite the limitation that the quantitative aspect and tissue protein binding of metabolites were not considered in this paper, the results of very low brain penetration of SAS as well as the significant levels of two metabolites (SP and Ac-SP) would be helpful to understand the new role of the metabolites in the brain. Further studies for these metabolites would be warranted for their CNS-related effects in vitro and in vivo.

## 4. Materials and Methods

### 4.1. Reagents and Chemicals

Sulfasalazine, verapamil, and deuterium oxide (D_2_O) were obtained from Merck and Sigma-Aldrich (Yong-in, Gyeonggi, Korea). Dimethyl sulfoxide (DMSO) and formic acid were all obtained from Daejung reagents (Siheung, Gyeonggi, Korea). HPLC grade methanol (MeOH) was acquired from Duksan reagents (Ansan, Gyeonggi, Korea). HPLC grade acetonitrile (ACN) and distilled water (DW) were all obtained from Samchun reagents (Gangnam, Seoul, Korea). All other chemicals were commercial products of either analytical grade or reagent grade, and no further purification was used

### 4.2. Preparation of Analytical Stock and Standards Solutions

Stock solutions of SAS was prepared in DMSO at 1 mg/mL concentrations and stored at 4 °C when not in use. Stock standards were freshly prepared in DMSO to 1.02, 3.05, 9.15, 27.4, 82.3, 247, 741, 2220, and 6670 ng/mL for SAS. The internal standards working solution using verapamil was prepared with ACN to a concentration of 100 ng/mL for pharmacokinetic study and 10 ng/mL for MetID study.

### 4.3. Sample Preparation

For pharmacokinetic study sample preparation, 20 µL of plasma samples were transferred to cluster tubes and mixed with 4 µL of stock standards for plasma matrix standard curve. There were 100 µL of internal standards solution in ACN added to each sample for extraction. The resulting solutions were vortex-mixed for 30 s and centrifuged at 13,000 rpm for 5 min to precipitate proteins in the matrix. The supernatant was three times diluted by distilled water and transferred to LC vial for LC-ESI-TOF-MS analysis.

There were 150 µL of brain homogenates transferred to 1.7 mL Eppendorf tubes including 150 µL of blank plasma and mixed with 30 µL of stock standards for brain surrogate-matrix standard curve. Then, 1 mL of internal standards solution in ACN was added to each sample for extraction. The result solutions were vortex-mixed for 30 s and centrifuged at 13,000 rpm for 5 min to precipitate protein in the matrix. The supernatant was transferred to clean 1.7 mL Eppendorf tubes and evaporated to dryness. The dried extract was reconstituted in 200 µL of 30% ACN in DW and transferred to LC vial for LC-ESI-TOF-MS analysis.

For MetID study sample preparation, plasma samples collected at 15, 30, 60, 120, and 240 min were pooled according to the Hamilton pooling method and the same Hamilton pooling method was applied to the brain homogenate samples collected at the same time and were transferred to 15 mL tubes [29,30,31]. Total volume of pooled plasma and brain homogenate samples were 1 mL and 3mL, respectively. After pooling, 3 and 9 mL of 50% MeOH in ACN was added to each plasma and brain surrogate-matrix for extraction, respectively. After vortexed for 1 min, the extraction solutions were centrifuged at 13000 rpm for 5 min. The supernatant was transferred to another tube and the evaporation and reconstitution processes using the 50% MeOH were conducted for MetID study.

### 4.4. LC-ESI-TOF-MS Condition

The LC-ESI-TOF-MS system for this experiment consisted of a chromatographic pump system (Shimadzu CBM-20A/LC-20AD, Shimadzu Scientific Instruments, Riverwood Dr, Columbia, SC, USA) and an auto-sampler system (Eksigent CTC HTS PAL, Sciex, Redwood City, CA, USA) equipped with a mass spectrometer (TripleTOF^TM^ 5600, Sciex, Redwood City, CA, USA). Chromatographic separation was performed on a reversed-phase C_18_ column (Phenomenex^®^ Kinetex XB-C18 column; 2.1 × 50 mm for bioanalytical sample quantification and 2.1 × 100 mm for MetID). A guard column was placed upstream of the analytical column. Mobile phase A (0.1% formic acid in distilled water) and mobile phase B (0.1% formic acid in acetonitrile) were used following an optimized gradient profile for the best separation of the analytes. The LC-gradient was optimized as follows: 3 min for the quantification (0–0.5 min, 10% B; 0.5–1.8 min, 10–95% B; 1.8–2.0 min, 95% B; 2.0–2.1 min, 95–10% B; and 2.1–3.0 min, 10% B with a flow rate of 0.4 mL/min), and 20 min for MetID (0–0.5 min, 5% B; 0.5–15 min, 5–40% B; 15–15.5 min, 40–95% B; 15.5–16.5 min, 95% B; 16.5–16.6 min, 95–5% B; and 16.6–20 min, 5% B with a flow rate of 0.3 mL/min).

In pharmacokinetic study, the curtain gas was 33 L/min, the gas source 1 and 2 were 50 psi, the ion spray voltage (ISVF) was set at 5500 V and the source temperature was 500 °C. The high resolution TOF full scan and two product ion scan for SAS and verapamil using single reaction monitoring at high resolution option mode was used for the PK sample analysis. Quantification was performed using the transitions *m*/*z* 399.1 > 381.1 (DP = 168 and CE = 22) for sulfasalazine and the transitions *m*/*z* 455.3 > 165.1 (DP = 125 and CE = 30) for verapamil, respectively.

In the MetID study, the curtain gas, ISVF and the source temperature were performed under identical experimental conditions. The high resolution TOF full scan and nine product ion scans for SAS and its known metabolites were performed. The following conditions were used to identify the SAS and its metabolites; TOF-MS scan (mass range: *m*/*z* 100–700, DP: 100 and CE: 10), product ion scan for SAS (DP: 168 and CE: 25). Other mass spectrometric conditions are summarized in Table 7.

### 4.5. Animal Study Design

All experimental protocols performed on mice were approved by the animal care institute from Chungnam National University (protocol no. CNU-01104). Male ICR mice were purchased from the Samtako Biokorea Co. (Gyeonggi, Korea) and housed in groups of 4~5 per cage with free access to standard rodent chow (labdiet 5L79, Orientbio, Korea).

SAS was administered to male ICR mice to evaluate the pharmacokinetics, brain-to-plasma coefficient (Kp_brain_), and the MetID of SAS after single dose of 10 mg/kg IV and 50 mg/kg PO administration. Animals were randomly assigned to two groups for five time points (*n* = 3), a total fifteen mice per each administration type. Body weights for the mice assigned to the study ranged from 28 to 30 g. SAS was dissolved in 20% DMSO, 20% PEG400 in DW for IV administration, and 30% DMSO, 20% PEG400 in DW for PO administration. Animals were sacrificed at 15, 30, 60, 120, and 240 min after dosing. At each time point, blood samples were first obtained using the heparinized tubes and centrifuged at 13,000 rpm for 5 min. After the 20~30 mL systemic perfusion with phosphate buffered saline (PBS), the brain was removed from the skull and then washed and homogenized using PBS in a ratio of 1:3 for tissue to buffer. The plasma and brain homogenates were placed in clean Eppendorf tubes and frozen at −20 °C until analysis.

### 4.6. H/D Exchange Study

H/D exchange is a well-established technique for studying structure, stability, folding dynamics, and intermolecular interactions in proteins in solution. The use of LC-ESI-TOF-MS equipped with an ESI source and deuterium oxide (D_2_O) as infusion solvent allows H/D exchange for compounds. Comparison of infusion method using 500 ng/mL of SAS dissolved in 50% H_2_O in ACN with 0.1% formic acid and 50% D_2_O in ACN with 0.1% formic acid were performed to elucidate CID pattern of SAS.

### 4.7. Data Analysis for Pharmacokinetic and MetID Study

The plasma and brain PK parameters (terminal half-life (T_1/2_), time to reach maximum concentration (T_max_), the area under the curve up to the last time point (AUC_last_)) were estimated from the mean concentrations at each time by non-compartmental analysis (NCA) using Phoenix WinNonlin^®^ Version 8.0.0 (Certara, Princeton, NJ, USA). The T_1/2_ and AUC_last_ were calculated using a linear trapezoidal linear interpolation method; T_max_ were observed values. The brain to plasma AUC ratios of the compounds (Kp_brain_) were calculated as follows:

brain to plasma AUC ratios (Kp_brain_) = AUC_last-brain_/AUC_last-plasma_ (1)

The AUC_last-brain_ and AUC_last-plasma_ were each AUC_last_ of the brain and plasma, respectively.

Data acquisition and LC-ESI-TOF-MS operation were conducted using Analyst^®^ TF Version 1.6 (Sciex). MultiQuant^®^ Version 2.1.1 (Sciex) was used for the peak integration of SAS for quantification. PeakView^®^ Version 2.2 and MetabolitePilot^TM^ Version 2.0.2 were used for the structural elucidation of SAS metabolites.

## Figures and Tables

**Table 1:** The inter-run assays results for sulfasalazine (*n* = 3).

Run	Nominal Concentration (ng/mL)	Calculated Concentration (ng/mL)	Mean Accuracy (%)	Precision (% CV)
Run 1	QC low [15.0]	14.3	95.1	10.0
	QC medium [165]	167	101	6.90
	QC high [1820]	2070	114	3.79
Run 2	QC low [15.0]	14.2	94.2	5.98
	QC medium [165]	160	97.1	1.69
	QC high [1820]	1950	107	1.10
Run 3	QC low [15.0]	16.3	108	4.95
	QC medium [165]	161	97.6	7.00
	QC high [1820]	1930	106	4.56

**Table 2:** The intra-run assays and results for sulfasalazine (*n* = 9).

Nominal Concentration (ng/mL)	Calculated Concentration (ng/mL)	Mean Accuracy (%)	Precision (% CV)
QC low [15.0]	14.9	99.2	8.71
QC medium [165]	163	98.7	5.11
QC high [1820]	1980	109	4.09

**Table 3:** The repeat injection of lower limit of quantification (LLOQ) results for sulfasalazine (*n* = 3).

Nominal Concentration (ng/mL)	Calculated Concentration (ng/mL)	Mean Accuracy (%)	Precision (% CV)
9.15	10.1	111	13.0

**Table 4:** The dilution quality control (QC) results for sulfasalazine (*n* = 3).

Dilution Factor	Nominal Concentration (ng/mL)	Calculated Concentration (ng/mL)	Mean Accuracy (%)	Precision (% CV)
×10	20,000	17,700	88.4	13.9

**Table 5:** Pharmacokinetic parameters of sulfasalazine from IV administration PK study (10 mg/kg).

Dosing Route	Matrix	Dose (mg/kg)	T_1/2_ (min)	C_max_ (ng/mL or ng/g)	AUC_last_ (min × ng/mL or min × ng/g)	Vd (mL/kg)	CL (mL/min/kg)	Brain to Plasma Ratio (%)
IV administration	Plasma	10	82	12,500	674,000	1740	14.8	1.26
	Brain		269	126	8460	−	−	

Cmax: observed maximum plasma concentration; Vd: Volume of distribution; CL: clearance.

**Table 6:** In vivo MetID results of sulfasalazine after PK study (IV: 10 mg/kg PO: 50 mg/kg).

Name	Formula	Exact *m*/*z*	Error (ppm)	Nominal Mass Change (Da)	RT (min)
SAS	C_18_H_1__5_N_4_O_5_S^+^	399.0763	−	−	13.84
SP	C_11_H_1__2_N_3_O_2_S^+^	250.0650	−7.2	−149	3.94
SP-OH	C_11_H_1__2_N_3_O_3_S^+^	266.0599	−6.4	−133	3.92
Ac-SP	C_1__3_H_1__4_N_3_O_3_S^+^	292.0756	−4.5	−107	5.23

RT: retention time (min).

**Table 7:** The mass spectrometric conditions for three sulfasalazine metabolites in high resolution product ion mode.

Metabolite	SP	SP-OH	Ac-SP
Mass Range (*m*/*z*)	50~500		
Product of (*m*/*z*)	250.1	266.1	292.1
DP (V)	100	100	100
CE (V)	30	30	30

SP: Sulfa pyridine; SP-OH: Hydroxy-sulfapyridine; Ac-SP: N-acetyl sulfapyridine.

## Data Availability

The data presented in this study are available on request from the corresponding author.

## Sample Availability

Samples of the compounds are not available from the authors.
